# Supplementary material for: Photodynamic Therapy with Nebulized Nanocurcumin on A549 Cells, Model Vessels, Macrophages and Beyond
Source: Pharmaceutics. 2022 Nov 29;14(12):2637. doi: 10.3390/pharmaceutics14122637 (PMC9781346; doi:10.3390/pharmaceutics14122637)
Supplement: Supplementary file 1 [file pharmaceutics-14-02637-s001.zip › pharmaceutics-2024871-supplementary.pdf]

Supplementary Information

Photodynamic Therapy with Nebulized Nanocurcumin on A549 Cells, Model Vessels, Macrophages and Beyond

S1. Irradiation device

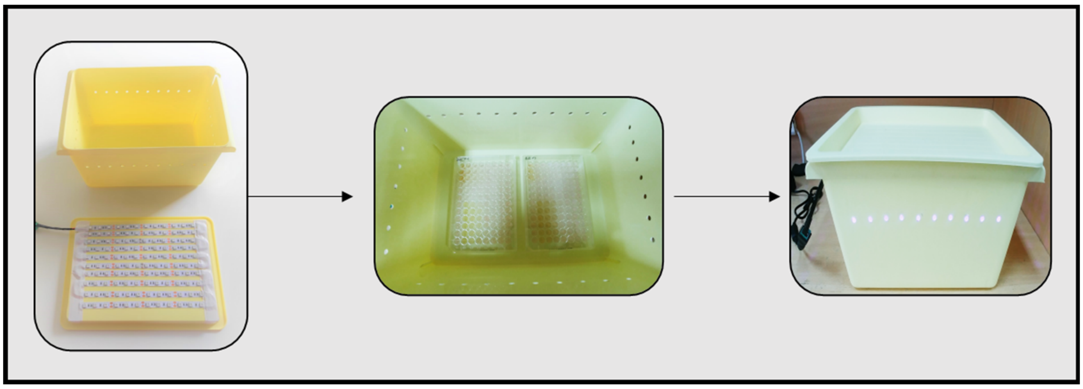

Figure S1. Home-made device with LED lights of 420 nm in the inner face of the lid, power of 30 mW/cm^2

S2. Structural features of nanovesicular CUR

Table S1. Structural features of nanovesicular CUR.

Formulation	CUR (µg/mL)	Total Lipids (mg/mL)	Mean Diameter (nm)	PdI
nATC	149 ± 81	15.0 ± 3.5	180 ± 40	0.24 ± 0.06
nLTC	155 ± 48	14.7 ± 2.9	226 ± 62	0.15 ± 0.08

Results are the media of 3 batches.

S3. Cytotoxicity of void nanovesicles and DMSO upon irradiation by MTT assay

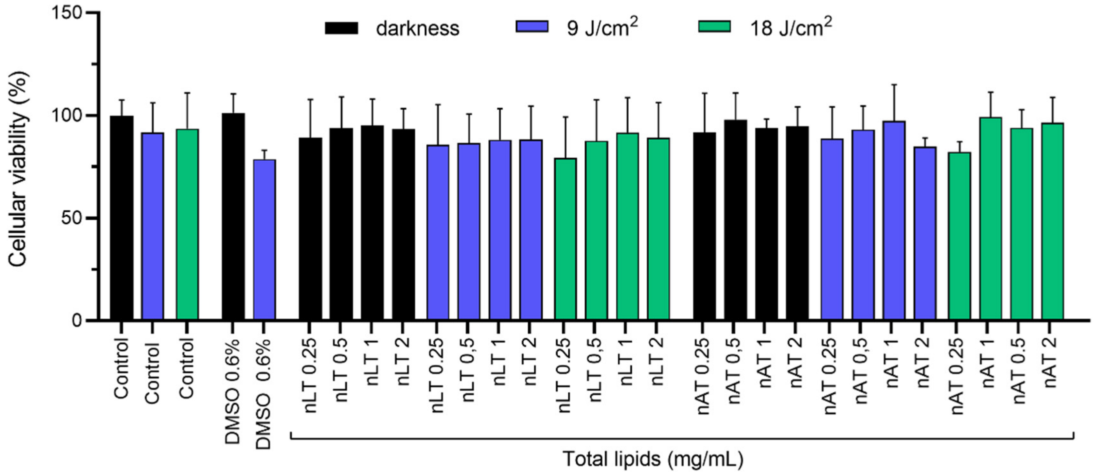

Figure S2. A549 cell line viability determined by MTT assay after 24 h incubation with nAT, nLT, free DMSO or MEM alone and irradiated with a fluence of 9 and 18 J/cm² or incubated in darkness.

S4. Early apoptosis determination by Annexin V and Propidium Iodide

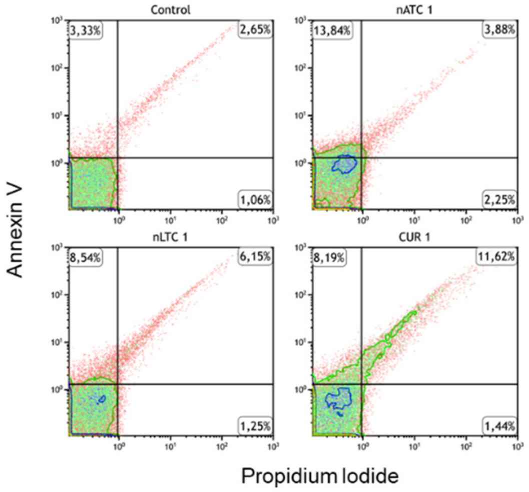

Figure S3. Density plot diagrams of apoptosis/necrosis assay on A549 cells. A549 cell line was incubated with nATC, nLTC or free CUR at 40 μM and irradiated (fluence of 9 J/cm²). 6 h after irradiation cells were incubated with Annexin V and PI.

S5. Global Score of Vascular damage

The lysis phenomena were scored according to the following scale:

Table S2. Lysis score according to vessel diameter[1]

Lysis level	Score
Without lysis	0
Quaternary vessels	1
Terciary vessels	2
Secondary vessels	3
Primary vessels	4

[1]The primary vessels are the main vessels, which branch out into secondary vessels, then tertiary vessels and finally quaternary vessels, which are the ones with the smallest diameter.

The haemorrhage phenomena were scored according to the following scale:

Table S3. Haemorrhage Score

Haemorrhage level	Score
Without haemorrhage	0
Mild	1
Moderate	2
Severe	3
Coagulation	4

Table S4. Time factor

Vascular damage onset time (h)	Time factor
2	4
4	2
6	1.5
8	1
